# Comparison of CD8^+^ T Cell Accumulation in the Brain During Human and Murine Cerebral Malaria

**DOI:** 10.3389/fimmu.2019.01747

**Published:** 2019-07-24

**Authors:** Valentina Barrera, Michael J. Haley, Patrick Strangward, Elizabeth Attree, Steve Kamiza, Karl B. Seydel, Terrie E. Taylor, Danny A. Milner, Alister G. Craig, Kevin N. Couper

**Affiliations:** ^1^Department of Eye and Vision Science, University of Liverpool, Liverpool, United Kingdom; ^2^Faculty of Biology, Medicine and Health, The Lydia Becker Institute of Immunology and Inflammation, University of Manchester, Manchester, United Kingdom; ^3^Department of Histopathology, College of Medicine, University of Malawi, Blantyre, Malawi; ^4^Department of Osteopathic Medical Specialties, College of Osteopathic Medicine, Michigan State University, East Lansing, MI, United States; ^5^Blantyre Malaria Project, University of Malawi College of Medicine, Blantyre, Malawi; ^6^Center for Global Health, American Society for Clinical Pathology, Chicago, IL, United States; ^7^Department of Tropical Disease Biology, Liverpool School of Tropical Medicine, Liverpool, United Kingdom

**Keywords:** cerebral malaria, CD8^+^ T cells, brain, *Plasmodium falciparum*, *Plasmodium berghei*

## Abstract

CD8^+^ T cells have been shown to play a critical role in the pathogenesis of experimental cerebral malaria (ECM) in mice, but their role in development of human cerebral malaria (HCM) remains unclear. Thus, in this study we have provided the first direct contrast of the accumulation of CD8^+^ T cells in the brain during HCM and ECM. HCM cases were from children who died of *Plasmodium falciparum* cerebral malaria at Queen Elizabeth Central Hospital (Malawi) between 2003 and 2010. ECM was induced by infecting C57BL/6J mice with *P. berghei* ANKA. We demonstrate similarities in the intracerebral CD8^+^ T cell responses in ECM and HCM, in particular an apparent shared choroid plexus—meningeal route of CD8^+^ T cell accumulation in the brain. Nevertheless, we also reveal some potentially important differences in compartmentalization of CD8^+^ T cells within the cerebrovascular bed in HCM and ECM.

## Introduction

Cerebral malaria (HCM) is a severe neurological complication of *Plasmodium falciparum* (*Pf*) infection that despite anti-malarial drug treatment often results in death or disability (1–4). Sequestration of parasitized red blood cells (pRBCs) within the cerebrovasculature is believed to be critical for the development of the syndrome in humans (3–6). However, the pathological processes downstream of pRBC sequestration that drive resultant neuropathology remain unclear (7). The *P. berghei* ANKA (*Pb* ANKA) murine model of experimental cerebral malaria (ECM) model has been extensively utilized to study the pathogenesis of HCM. In this model, intracerebral CD8^+^ T cells play a major role in disruption of the blood brain barrier and formation of cerebral pathology (8, 9). Whether CD8^+^ T cells contribute to the development of HCM is unknown and has remained a matter of significant debate in the malaria community (10).

To address whether CD8^+^ T cells may play comparable roles during HCM and ECM we have quantified, by histopathological investigation, CD8^+^ T cells in human and murine brains during HCM and ECM, respectively. We examined CD8^+^ T cell compartmentalization in the cortical cerebrovasculature, leptomeninges and choroid plexus (CP) to account for the three different routes through which T cells can enter and accumulate in the brain (11): via the blood brain barrier (BBB), the blood-meningeal barrier (BMB) or the blood-cerebrospinal fluid barrier (BCSFB), respectively.

## Materials and Methods

### Ethics

The HCM study was approved by research ethics committee at the University of Malawi College of Medicine P. 11/07/593, Michigan State University, Liverpool School of Tropical Medicine (protocol 12.29) and the Royal Liverpool and Broadgreen University Hospital Trust no. 3690. All research was performed in accordance with the Declaration of Helsinki. The ECM study was approved following local ethical review by the University of Manchester Animal Procedures and Ethics Committees and was performed in strict accordance with the U. K. Home Office Animals (Scientific Procedures) Act 1986 (approved H.O. Project License P8829D3B4).

### HCM Study Setting

We studied cerebral pathology of a total of 17 Malawian children by accessing a unique archive of tissue blocks obtained from a prospective cohort which was recruited between 1996 and 2011 in Blantyre (5, 12, 13). Cases used in this study were recruited between 2003 and 2010. Autopsies were performed with parents' or guardians' consent as quickly after death as possible in the morgue of the Queen Elizabeth Central Hospital in Blantyre, Malawi. Seven children died of clinically and histopathologically defined CM [2 cases of CM1 and 5 cases of CM2; for a detailed description of CM classification see (12)], with Blantyre coma score of ≤2, peripheral *P. falciparum* parasitemia, no other obvious cause of coma i.e., hypoglycemia, post-ictal state, or meningitis, and pRBC sequestration within cerebral vessels. CM diagnosis was confirmed by presence of malarial retinopathy (12, 14). We also examined the brains of 5 CM3 cases, which were initially classified as clinical HCM but which were found to have alternative causes of death (including pneumonia and intracerebral hematoma), 2 CM7 cases (Non-malarial encephalopathy, *Salmonella* sepsis cause of death), 2 CM9 cases and 1 CM11 case (other non-infectious causes of death, but with incidental parasitemia).

### Immunohistochemistry for Human Tissue

At autopsy, the brain was dissected and corresponding, representative sections from the frontal lobe and choroid plexus were fixed in 10% neutral buffered formalin, processed, embedded in paraffin, and sectioned at 3–4 microns. Sections were stained via the indirect immunoperoxidase method for CD8 as previously described (5). Briefly, sections were deparaffinized and rehydrated before microwave antigen retrieval was performed in Tris EDTA pH9 buffer. After blocking endogenous peroxidase, sections were incubated with 5% bovine serum albumin in Tris–Tween 20 buffer at room temperature for 30 min and then incubated with a 1:80 dilution of stock CD8 antibody (C8/144B, mouse monoclonal; DAKO) overnight at 4°C. After washing in Tris–Tween 20 buffer, sections were incubated for 1 h with a 1:500 dilution of goat anti-mouse conjugated to horseradish peroxidase, before being incubated in 3,3′-diaminobenzidine for 5 min, and counterstained with hematoxylin.

### Mice and Infections

Six female 8–10 weeks old C57BL/6 mice were purchased from Charles River and used for infection, and five C57BL/6 mice were used as naïve controls. All mice were maintained in individually ventilated cages at the University of Manchester. Cryopreserved *Pb* ANKA parasites clone cl15cy1 (15) were thawed and passaged once through C57BL/6 mice before being used to infect experimental animals. Animals were infected via intravenous injection of 1 × 10^4^ parasitized red blood cells (pRBCs). The development of ECM was assessed using a well-established clinical scale (15): 1 = no signs; 2 = ruffled fur and/or abnormal posture; 3 = lethargy; 4 = reduced responsiveness to stimulation and/or ataxia and/or respiratory distress/hyperventilation; 5 = prostration and/or paralysis and/or convulsions. Stages 4–5 were classified as ECM. *P. berghei* ANKA infected mice progressed through stages 1–4 on day 6 of infection and were euthanized when they reached stage 5 on day 7 of infection, following a course of infection we have described previously (5).

### Immunohistochemistry for Mouse Tissue

*Pb* ANKA infected mice were killed by CO_2_, brains removed and immerse fixed in 4% paraformaldehyde. Brains were then processed for paraffin embedding, and sectioned at 4 μm using a Leica RM 2155 Microtome (Leica Microsystems Ltd., Germany). Sections were deparaffinized and rehydrated before undergoing heat-mediated antigen retrieval (Tris EDTA pH 9). After blocking endogenous peroxidase, sections were incubated with blocking solution (0.3% Triton-X100 and 1% bovine serum albumin in PBS) at room temperature for 1 h and then incubated with a 1:2,000 dilution of stock CD8 antibody (EPR20305, rabbit monoclonal; Abcam) overnight at 4°C. Sections were then incubated in a 1:500 dilution of a biotinylated anti-rabbit antibody (Vector), followed by a signal amplification step using a VECTASTAIN Elite ABC HRP Kit (Vector) for 1 h. Sections were then incubated in 3,3′-diaminobenzidine for 5 min, and counterstained with hematoxylin.

### Microscopical Topographical Quantification of CD8^+^ Cells

For mouse tissue, images were collected on an Olympus BX63 upright microscope using a 20×/0.75 UApo/340 objective and captured and white-balanced using a DP80 camera (Olympus) in color mode through CellSens Dimension v1.16 (Olympus). For human tissue, images were acquired on a slide-scanner microscope (Leica) using a 20×/0.30 Plan Achromat objective (Zeiss). Snapshots of the slide-scans were taken using Aperio ImageScope (Leica). Images were then processed and analyzed using Fiji ImageJ (http://imagej.net/Fiji/Downloads). For mice, 8 fields per region (cortex and choroid) were quantified, for a total area of 2.9 mm^2^. For each human case, four 4 mm^2^ fields were quantified in each region, for a total of 16 mm^2^ per region. Several parameters were measured to assess CD8^+^ T cell accumulation in each region: absolute number of CD8^+^ T cells, proportion of vessels with CD8^+^ T cells, proportion of CD8^+^ T cells that have transmigrated across the vascular lumen. In human cases both parasitized and non-parasitized vessels were examined. If detected, CD8^+^ monocytes were not included in counts, and were distinguishable from lymphocytes due to their clearly defined kidney-shaped nuclei. Scoring was performed blinded to CM status by two independent observers, with <10% intra-observer variation found.

### Statistical Analyses

All statistical analyses were performed using GraphPad PRISM (GraphPad Software). Normality was assessed with Shapiro-Wilk tests and equal variances with Brown–Forsythe tests. Individual tests applied are detailed in figure legends.

## Results

**Figure 1:**
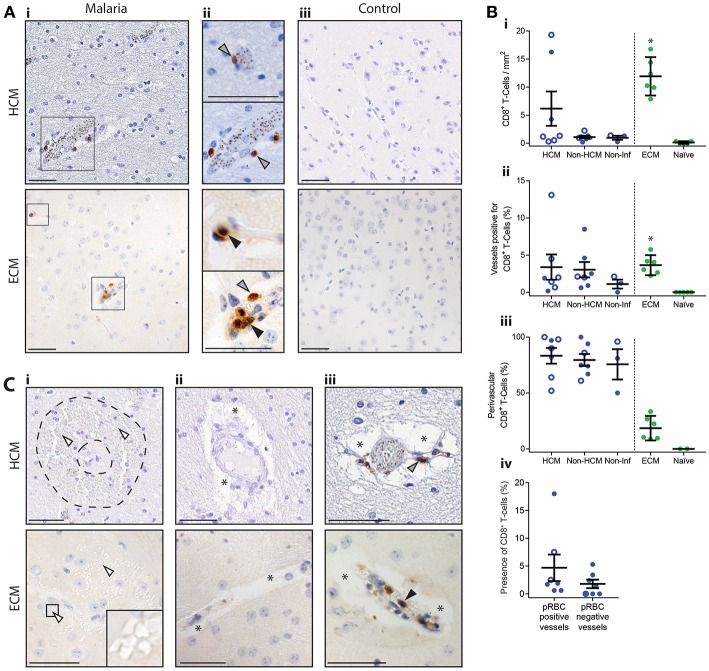
Distribution of intracerebral CD8^+^ T cells in the cortex in HCM and ECM. The presence of intracerebral CD8^+^ T cells was assessed by immunohistochemistry in fatal HCM cases, in C57BL/6 mice that developed late-stage ECM after *Pb* ANKA infection, and in appropriate controls. **(A)** Representative images from HCM and ECM cases showing (Ai) low magnification and (Aii) high magnification identification of both luminal (black arrows) and perivascular (gray arrows) CD8^+^ T cells, in capillaries and large caliber vessels. (Aiii) Representative images showing lack of CD8^+^ T cells in brains of HCM controls (CM9) and naïve mice. **(B)** Several parameters of CD8^+^ T cell accumulation were quantified: (Bi) absolute numbers of CD8^+^ T cells per mm^2^, (Bii) percentage of total vessels with CD8^+^ T cells (either luminal or perivascular), (Biii) percentage of CD8^+^ T cells that were perivascular, (Biv) percentage of vessels in HCM cases with CD8^+^ T cells separated into vessels with and without parasitized red blood cells (pRBCs). HCM = CM1 (filled circles) and CM2 (open circles) cases, Non-HCM = CM3 (filled circles) and CM7 (open circles), Non-Inf = CM9 (filled circles) and CM11 (open circles). **(C)** Representative images from HCM cases and ECM samples showing: (Ci) intracerebral hemorrhage (white arrows indicate red blood cells, note classical ring hemorrhage in HCM, demarcated by dotted outline), areas of vasogenic edema (asterisks) without (Cii) and with CD8^+^ T cells (Ciii). Scale bars = 50 μm. *p < 0.05. Human cases were compared by one-way ANOVA with Holm-Sidak's multiple comparisons test, ECM was compared to naïve by Student's *t*-test, and presence of CD8^+^ T cells in pRBC positive and negative vessels was compared by Student's *t*-test.

The comparative histopathology investigation revealed very few CD8^+^ T cells within the cortex in both HCM and ECM cases (Figures 1A,B); however, numbers in ECM were on average double those in HCM (Figure 1Bi). Although the density of CD8^+^ T cells in the cortex was higher in subjects who had confirmed fatal HCM (CM1 and CM2), compared with control human non-CM malaria cases (CM3 and CM7), or non-infectious control cases (CM9 and CM11) (Figures 1Ai,iii,Bi), within the number of samples examined, the difference was not statistically significant. In contrast, CD8^+^ T cell accumulation in the cortex was significantly higher in ECM than in naïve mice (Figures 1Ai,iii,Bi).

In both ECM and HCM CD8^+^ T cells were observed most frequently singular and associated with capillaries (classified by measuring vessel diameter) (Figure 1Aii). However, multiple CD8^+^ T cells were occasionally found associated with non-capillary (larger-caliber) vessels, which were often packed with RBCs and leukocytes (Figure 1Aii). Interestingly, on average, 4% of cortical blood vessels in HCM and ECM brains were positive for CD8^+^ T cells (Figure 1Bii). The increased density of CD8^+^ T cells in ECM compared with HCM was, therefore, not because CD8^+^ T cells were associated with a higher percentage of vessels, but was because there was a greater frequency of packed vessels with clusters of CD8^+^ T cells in ECM. In HCM, blood vessel-associated CD8^+^ T cells in the cortex were predominantly perivascular (i.e. had crossed the BBB but not passed the glial limitans or penetrated into parenchyma), whereas in ECM they were predominantly luminal (Figures 1Aii,Biii). There was no significant difference in the percentage of pRBC positive and pRBC negative vessels associated with CD8^+^ T cells during HCM, indicating that pRBC presence does not promote or impede CD8^+^ T cell accumulation during HCM (Figure 1Aiv). Notably, in neither the HCM nor ECM cases examined were spatial relationships between CD8^+^ T cells and pathological features apparent: Perivascular edema was observed in vessels with and without CD8^+^ T cells, and there was a lack of CD8^+^ T cells at sites of hemorrhage (Figure 1C).

**Figure 2:**
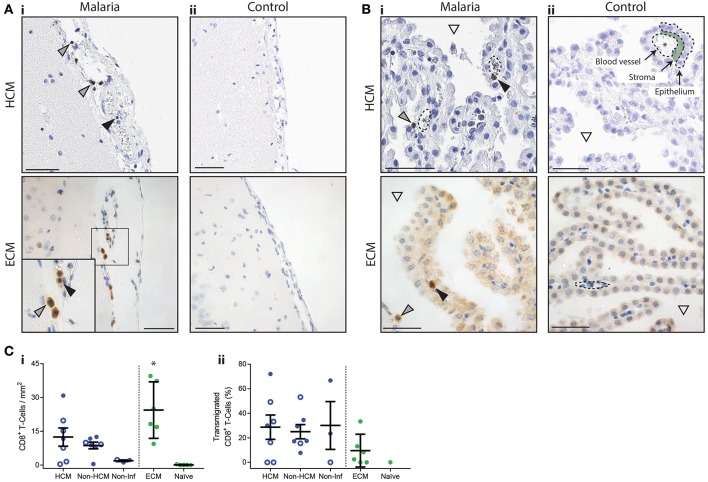
Distribution of CD8^+^ T cells in the leptomeninges and choroid plexus in HCM and ECM. The presence of CD8^+^ T cells in the leptomeninges and choroid plexus was assessed by immunohistochemistry in fatal HCM cases, in C57BL/6 mice that developed late-stage ECM after *Pb* ANKA infection, and in appropriate controls. **(A)** Representative images of the leptomeninges from HCM and ECM cases showing CD8^+^ T cells in the lumen of pial vessels (black arrows), and in the subarachnoid space (gray arrows). **(B)** Representative images of the choroid plexus from HCM and ECM cases showing CD8^+^ T cells in the stroma or lumen (asterisks) of blood vessels (black arrows), or that have transmigrated into the ventricle (gray arrows; white triangle indicates CSF-filled ventricle). **(C)** (Ci) Absolute numbers of CD8^+^ T cells per mm^2^ of choroid plexus; (Cii) percentage of CD8^+^ T cells in the choroid plexus that had transmigrated into the ventricle. HCM = CM1 (filled circles) and CM2 (open circles) cases, Non-HCM = CM3 (filled circles) and CM7 (open circles), Non-Inf = CM9 (filled circles) and CM11 (open circles). Scale bars = 50 μm. *p < 0.05. Human cases were compared by one-way ANOVA with Holm-Sidak's multiple comparisons test, ECM was compared to naïve by Students *t*-test.

CD8^+^ T cells were observed throughout the leptomeninges in both HCM and ECM samples (Figure 2A). CD8^+^ T cells were found in the lumen of pial vessels, and within the cerebrospinal fluid (CSF)-filled subarachnoid space (SAS), in qualitatively higher numbers than in the cortex (Figure 2A). The choroid plexus has been shown to be a major route for T cell trafficking via the ventricles to the leptomeninges (11, 16). In agreement with this there was a significant accumulation of CD8^+^ T cells in the CP during ECM (compared with uninfected controls) and, within the small number of cases examined, there was a trend toward higher numbers of CD8^+^ T cells in the CP during HCM, compared with non-infectious controls (Figures 2B,Ci). The density of CD8^+^ T cells was also significantly higher in the CP than in the cortex in ECM samples (11.9 in cortex, 24.4 in CP, *p* = 0.039), and trended higher in HCM cases (6.2 in cortex, 12.4 in CP, *p* = 0.076). In both ECM and HCM, CD8^+^ T cells were observed in the lumen of CP blood vessels, but were also found in the stroma, or to have transmigrated into the ventricles (Figures 2B,Cii; black or gray arrows, respectively). Thus, our results suggest a comparable choroid plexus–meningeal route of CD8^+^ T cell entry into the superficial areas of the brain may be present in both ECM and HCM.

## Discussion

In this study we have confirmed the presence of CD8^+^ T cells in both HCM and ECM brain pathology, but that very few CD8^+^ T cells are required in the cerebrovasculature for development of ECM, where they play a known important role in disease pathogenesis (8, 9). This observation redefines the expectations of the numbers of CD8^+^ T cells that should be seen in HCM. Whilst we only examined CD8^+^ T cell accumulation within the cortex in HCM and ECM brains within this study, we have previously shown that CD8^+^ T cell numbers are highest in the cortex during ECM compared with other brain regions, including the brain stem and olfactory bulbs. Moreover, there is also a high level of pathology within the cortex, compared with other brain regions, during both HCM and ECM (5, 15). CD8^+^ T cell numbers were heterogeneous in the different HCM cases, which may relate to the fact CM1 and CM2 are distinct syndromes (5), as well as temporal differences in course of infection and timing of death of the different patients. The assessment of additional CM1 and CM2 cases would be required to definitively address the contribution of these parameters in controlling CD8^+^ T cell numbers within the brain during HCM. Whether the administration of anti-malarial drugs (and the duration from treatment) influenced intracerebral CD8^+^ T cell numbers in HCM cases, or affected the comparison with ECM samples, where mice did not receive any treatment, also requires further investigation. Nevertheless, overall, from the number of HCM cases examined, our results suggest that the density of CD8^+^ T cells in the brain during ECM may be higher than in HCM CM1 and CM2 cases.

We observed CD8^+^ T cells in a comparably low percentage of vessels during ECM and HCM. Whilst this may suggest that CD8^+^ T cells equally influence the cerebrovascular network in ECM and HCM, CD8^+^ T cells were primarily luminal in the cortex in ECM, as previously reported (15, 17). In contrast, CD8^+^ T cells were predominantly perivascular within the brain in HCM. The reason for the difference in compartmentalization of CD8^+^ T cells between ECM and HCM is unclear as activation of cerebral endothelial cells appears similar in HCM and ECM (7, 8, 18), and both human and murine brain endothelial cells seem able to phagocytose and present *Plasmodium* spp. antigens for recognition by CD8^+^ T cells (19, 20). Further work will thus be required to investigate if CD8^+^ T cells interact differently with the vascular endothelial cells during ECM and HCM, and if the contrasting compartmentalization means CD8^+^ T cells have differing importance during the HCM and ECM syndromes.

Our results suggest a comparable choroid plexus–meningeal route of CD8^+^ T cell entry into the superficial areas of the brain may be present in both ECM and HCM. In qualitative analyses the density of CD8^+^ T cells was higher in the leptomeninges than in the cortex during HCM and ECM, and the numbers of CD8^+^ T cells in the CP was significantly higher and trended higher than in control samples during ECM and HCM, respectively. The CP is an important entry point into the CSF containing SAS where T cell immunosurveillance of the CNS occurs (11, 16). Thus, activated T cells accumulate in the SAS and are exposed to material in the interstitial fluid drained to this site via the glymphatics system (21). Whilst, the relative importance of CD8^+^ T cells located within the choroid plexus and leptomeninges in the pathogeneses of ECM and HCM is unknown, it has recently been shown that meningeal inflammation can lead to CXCL10 production that signals inward to the brain parenchyma affecting neuronal function (22). CXCL10 is involved in the pathogenesis of ECM, including through promoting T cell migration and stabilizing T cell adhesion to brain endothelial cells (23, 24), and has been associated with HCM development (25). Therefore, it is possible that the accumulation of CD8^+^ T cells within the choroid plexus and leptomeninges may trigger inward transmission of inflammatory signals, potentially through the CSF-filled perivascular spaces, contributing to malaria-induced cerebral pathology. The presence of CD8^+^ T cells in the CP in CM3 and CM7 cases may be because these patients died of conditions with a known inflammatory element (e.g., non-malarial encephalopathy). In support of this, CD8^+^ T cells were rarely found in the CP of patients with non-infectious causes of death, or in naïve mice.

In conclusion, in this first direct comparative study we demonstrate CD8^+^ T cells are observed in very low numbers in both ECM and HCM, and that CD8^+^ T cells are associated with a comparable proportion of blood vessels in HCM and ECM. Nevertheless, there are important differences in the compartmentalization of CD8^+^ T cells in HCM and ECM, which may influence the relative role of the cells in the two syndromes. Further investigations into the impact of CD8^+^ T cells, and other immunological mediators, in the leptomeninges to malaria-induced encephalopathy are warranted.

## Data Availability

All datasets generated for this study are included in the manuscript and/or the supplementary files.

## Ethics Statement

The HCM study was approved by research ethics committee at the University of Malawi College of Medicine P. 11/07/593, Michigan State University, Liverpool School of Tropical Medicine (protocol 12.29) and the Royal Liverpool and Broadgreen University Hospital Trust no. 3690. All research was performed in accordance with the Declaration of Helsinki. The ECM study was approved following local ethical review by the University of Manchester Animal Procedures and Ethics Committees and was performed in strict accordance with the U. K. Home Office Animals (Scientific Procedures) Act 1986 (approved H.O. Project License P8829D3B4).

## Author Contributions

MH, PS, and KC: conceptualization. VB, MH, PS, and DM: methodology. VB, MH, and PS: investigation. VB, MH, and EA: formal analysis. MH, PS, and KC: writing—original draft. VB, MH, AC, and KC: writing—review and editing. KC: funding acquisition. KS, SK, TT, and DM: resources. AC and KC: supervision. MH: visualization.

### Conflict of Interest Statement

The authors declare that the research was conducted in the absence of any commercial or financial relationships that could be construed as a potential conflict of interest.

## References

[1] World Malaria Report 2018. World Health Organisation. Available online at: http://www.who.int/malaria/publications/world-malaria-report-2018/en/

[2] Langfitt JT Mcdermott MP Brim R Mboma S Potchen MJ Kampondeni SD. Neurodevelopmental impairments 1 year after cerebral malaria. Pediatrics. (2019) 143:e20181026. 10.1542/peds.2018-1026 30696757

[3] Riggle BA Miller LH Pierce SK. Do we know enough to find an adjunctive therapy for cerebral malaria in African children? F1000Res. (2017) 6:2039. 10.12688/f1000research.12401.1 29250318 PMC5701444

[4] Wassmer SC Grau GE. Severe malaria: what's new on the pathogenesis front? Int J Parasitol. (2017) 47:145–52. 10.1016/j.ijpara.2016.08.002 27670365 PMC5285481

[5] Dorovini-Zis K Schmidt K Huynh H Fu W Whitten RO Milner D. The neuropathology of fatal cerebral malaria in Malawian children. Am J Pathol. (2011) 178:2146–58. 10.1016/j.ajpath.2011.01.016 21514429 PMC3081150

[6] White NJ Turner GD Day NP Dondorp AM. Lethal malaria: Marchiafava and Bignami were right. J Infect Dis. (2013) 208:192–8. 10.1093/infdis/jit116 23585685 PMC3685223

[7] Storm J Craig AG. Pathogenesis of cerebral malaria–inflammation and cytoadherence. Front Cell Infect Microbiol. (2014) 4:100. 10.3389/fcimb.2014.00100 25120958 PMC4114466

[8] Ghazanfari N Mueller SN Heath WR. Cerebral malaria in mouse and man. Front Immunol. (2018) 9:2016. 10.3389/fimmu.2018.02016 30250468 PMC6139318

[9] Howland SW Claser C Poh CM Gun SY Renia L. Pathogenic CD8^+^ T cells in experimental cerebral malaria. Semin Immunopathol. (2015) 37:221–31. 10.1007/s00281-015-0476-6 25772948

[10] White NJ Turner GD Medana IM Dondorp AM Day NP. The murine cerebral malaria phenomenon. Trends Parasitol. (2010) 26:11–5. 10.1016/j.pt.2009.10.007 19932638 PMC2807032

[11] Engelhardt B Vajkoczy P Weller RO. The movers and shapers in immune privilege of the CNS. Nat Immunol. (2017) 18:123–31. 10.1038/ni.3666 28092374

[12] Taylor TE Fu WJ Carr RA Whitten RO Mueller JS Fosiko NG. Differentiating the pathologies of cerebral malaria by postmortem parasite counts. Nat Med. (2004) 10:143–5. 10.1038/nm986 14745442

[13] Milner DA Jr Valim C Carr RA Chandak PB Fosiko NG Whitten R. A histological method for quantifying Plasmodium falciparum in the brain in fatal paediatric cerebral malaria. Malar J. (2013) 12:191. 10.1186/1475-2875-12-191 23758807 PMC3701562

[14] Barrera V Hiscott PS Craig AG White VA Milner DA Beare NA. Severity of retinopathy parallels the degree of parasite sequestration in the eyes and brains of Malawian children with fatal cerebral malaria. J Infect Dis. (2015) 211:1977–86. 10.1093/infdis/jiu592 25351204 PMC4442623

[15] Strangward P Haley MJ Shaw TN Schwartz JM Greig R Mironov A. A quantitative brain map of experimental cerebral malaria pathology. PLoS Pathog. (2017) 13:e1006267. 10.1371/journal.ppat.1006267 28273147 PMC5358898

[16] Baruch K Schwartz M. CNS-specific T cells shape brain function via the choroid plexus. Brain Behav Immun. (2013) 34:11–6. 10.1016/j.bbi.2013.04.002 23597431

[17] Swanson PA II Hart GT Russo MV Nayak D Yazew T Pena M. CD8^+^ T cells induce fatal brainstem pathology during cerebral malaria via luminal antigen-specific engagement of brain vasculature. PLoS Pathog. (2016) 12:e1006022. 10.1371/journal.ppat.1006022 27907215 PMC5131904

[18] Turner GD Morrison H Jones M Davis TM Looareesuwan S Buley ID. An immunohistochemical study of the pathology of fatal malaria. Evidence for widespread endothelial activation and a potential role for intercellular adhesion molecule-1 in cerebral sequestration. Am J Pathol. (1994) 145:1057–69. 7526692 PMC1887431

[19] Howland SW Poh CM Renia L. Activated brain endothelial cells cross-present malaria antigen. PLoS Pathog. (2015) 11:e1004963. 10.1371/journal.ppat.1004963 26046849 PMC4457820

[20] Jambou R Combes V Jambou MJ Weksler BB Couraud PO Grau GE. Plasmodium falciparum adhesion on human brain microvascular endothelial cells involves transmigration-like cup formation and induces opening of intercellular junctions. PLoS Pathog. (2010) 6:e1001021. 10.1371/journal.ppat.1001021 20686652 PMC2912387

[21] Kipnis J. Multifaceted interactions between adaptive immunity and the central nervous system. Science. (2016) 353:766–71. 10.1126/science.aag2638 27540163 PMC5590839

[22] Blank T Detje CN Spiess A Hagemeyer N Brendecke SM Wolfart J. Brain endothelial- and epithelial-specific interferon receptor chain 1 drives virus-induced sickness behavior and cognitive impairment. Immunity. (2016) 44:901–12. 10.1016/j.immuni.2016.04.005 27096319

[23] Campanella GS Tager AM El Khoury JK Thomas SY Abrazinski TA Manice LA. Chemokine receptor CXCR3 and its ligands CXCL9 and CXCL10 are required for the development of murine cerebral malaria. Proc Natl Acad Sci USA. (2008) 105:4814–9. 10.1073/pnas.0801544105 18347328 PMC2290783

[24] Sorensen EW Lian J Ozga AJ Miyabe Y Ji SW Bromley SK. CXCL10 stabilizes T cell-brain endothelial cell adhesion leading to the induction of cerebral malaria. JCI Insight. (2018) 3:98911. 10.1172/jci.insight.98911 29669942 PMC5931132

[25] Dunst J Kamena F Matuschewski K. Cytokines and chemokines in cerebral malaria pathogenesis. Front Cell Infect Microbiol. (2017) 7:324. 10.3389/fcimb.2017.00324 28775960 PMC5517394

